# Reduced Expression of GABA_*A*_ Receptor Alpha2 Subunit Is Associated With Disinhibition of DYT-THAP1 Dystonia Patient-Derived Striatal Medium Spiny Neurons

**DOI:** 10.3389/fcell.2021.650586

**Published:** 2021-05-21

**Authors:** Selma Staege, Anna Kutschenko, Hauke Baumann, Hannes Glaß, Lisa Henkel, Thomas Gschwendtberger, Norman Kalmbach, Martin Klietz, Andreas Hermann, Katja Lohmann, Philip Seibler, Florian Wegner

**Affiliations:** ^1^Department of Neurology, Hannover Medical School, Hanover, Germany; ^2^Center for Systems Neuroscience, Hanover, Germany; ^3^Institute of Neurogenetics, University of Lübeck, Lübeck, Germany; ^4^Translational Neurodegeneration Section “Albrecht-Kossel”, Department of Neurology, University of Rostock, Rostock, Germany; ^5^German Center for Neurodegenerative Diseases Rostock/Greifswald, Rostock, Germany

**Keywords:** DYT-THAP1, genetic dystonia, induced pluripotent stem cells, striatal medium spiny neurons, GABAA receptor, calcium dynamics, patch-clamp electrophysiology

## Abstract

DYT-THAP1 dystonia (formerly DYT6) is an adolescent-onset dystonia characterized by involuntary muscle contractions usually involving the upper body. It is caused by mutations in the gene *THAP1* encoding for the transcription factor Thanatos-associated protein (THAP) domain containing apoptosis-associated protein 1 and inherited in an autosomal-dominant manner with reduced penetrance. Alterations in the development of striatal neuronal projections and synaptic function are known from transgenic mice models. To investigate pathogenetic mechanisms, human induced pluripotent stem cell (iPSC)-derived medium spiny neurons (MSNs) from two patients and one family member with reduced penetrance carrying a mutation in the gene *THAP1* (c.474delA and c.38G > A) were functionally characterized in comparison to healthy controls. Calcium imaging and quantitative PCR analysis revealed significantly lower Ca^2+^ amplitudes upon GABA applications and a marked downregulation of the gene encoding the GABA_*A*_ receptor alpha2 subunit in THAP1 MSNs indicating a decreased GABAergic transmission. Whole-cell patch-clamp recordings showed a significantly lower frequency of miniature postsynaptic currents (mPSCs), whereas the frequency of spontaneous action potentials (APs) was elevated in THAP1 MSNs suggesting that decreased synaptic activity might have resulted in enhanced generation of APs. Our molecular and functional data indicate that a reduced expression of GABA_*A*_ receptor alpha2 subunit could eventually lead to limited GABAergic synaptic transmission, neuronal disinhibition, and hyperexcitability of THAP1 MSNs. These data give pathophysiological insight and may contribute to the development of novel treatment strategies for DYT-THAP1 dystonia.

## Introduction

DYT-THAP1 dystonia (previously referred to as DYT6) is an adolescent-onset dystonia with a mixed phenotype caused by mutations in the gene *THAP1* encoding for Thanatos-associated protein (THAP) domain containing apoptosis-associated protein 1. It is inherited in an autosomal-dominant manner with reduced penetrance (Djarmati et al., 2009; Fuchs et al., 2009; Albanese et al., 2013; Marras et al., 2016; Lohmann and Klein, 2017). DYT-THAP1 dystonia is characterized by involuntary muscle contractions of the head/neck, occasionally the arm and leg, but mostly involving the upper body. However, the clinical phenotype of DYT-THAP1 dystonia can vary from focal dystonia and also spread to generalized dystonia. Speech is usually affected due to laryngeal dystonia (Djarmati et al., 2009; Fuchs et al., 2009). Currently, only symptomatic treatments (systemic pharmacological therapy, e.g., with anticholinergic or GABAergic drugs, intramuscular botulinum toxin injection, and deep brain stimulation) are available (Thenganatt and Jankovic, 2014; Brüggemann et al., 2015), however, there is urgent need for disease-modifying therapies.

Structural and functional neuroimaging demonstrates abnormalities in cerebello-thalamo-cortical and cortico-striato-pallido-thalamo-cortical pathways (Niethammer et al., 2011). Further, genetically engineered mice with heterozygote *Thap1* mutations display structural abnormalities of the deep cerebellar nuclei and deficits on motor tasks without overt dystonia (Ruiz et al, 2015). Mice with loss of *Thap1* in glial and neuronal precursors show alterations in synaptic transmission and derangements in cerebellar and basal ganglia circuitry (Frederick et al., 2019).

To date, more than 80 different disease-causing mutations in *THAP1* are known, including missense, non-sense, and frameshift mutations (Blanchard et al., 2011; Lohmann et al., 2012)^1^. *THAP1* encodes a ubiquitously expressed zinc-finger transcription factor with DNA binding and protein-interaction domains (Campagne et al., 2010; Mazars et al., 2010) that plays a role in endothelial cell proliferation (Cayrol et al., 2007) and proapoptotic processes (Roussigne et al., 2003). Due to posttranslational modification, several *THAP1* splice variants exist, and a neuronal species was described previously assuming the disease phenotype to be restricted to the central nervous system (Ortiz-Virumbrales et al., 2014). It is shown that mutations in *THAP1* often lead to dysregulations in the development of neuronal projections and mitochondrial and synaptic function (Zakirova et al., 2018). The THAP1 protein binds to the THAP1-binding sequence (THABS), which is part of the *THAP1* promoter (Erogullari et al., 2014) as well as of the promotor of *TOR1A* that is the mutated gene of DYT-TOR1A dystonia (formerly DYT1; Gavarini et al., 2010; Kaiser et al., 2010). Therefore, *THAP1* is able to autoregulate its own expression (Erogullari et al., 2014). It is known from a mouse model of DYT-TOR1A dystonia that the *TOR1A* mutation leads to a change in the calcium dynamics and to an impairment of striatal D2 receptor-dependent control of GABAergic activity resulting in an increased GABAergic transmission in medium spiny neurons and fast-spiking interneurons (Sciamanna et al., 2009; Iwabuchi et al., 2013). However, further molecular and cellular functions as results of mutations in *THAP1* causing dystonia remain largely unknown.

In this study, we investigate the functional phenotype of striatal medium spiny neurons (MSNs) differentiated from DYT-THAP1 patient-derived induced pluripotent stem cells (iPSCs) and healthy controls. The generation and characterization of DYT-THAP1 iPSCs was published recently (Baumann et al., 2018). DYT-THAP1 iPSCs were derived from two affected patients and one family member with reduced penetrance (Zittel et al., 2010) carrying a frameshift mutation p.Lys158Asnfs^∗^23 manifesting in writer’s dystonia followed by laryngeal dystonia and a missense mutation p.Arg13His leading to generalized dystonia. The healthy control–derived iPSCs were also previously analyzed (Japtok et al., 2015; Glaß et al., 2018). For efficient differentiation of iPSCs into a large amount of MSNs, we adapted two of our published differentiation protocols (Stanslowsky et al., 2016; Capetian et al., 2018). We used calcium imaging and whole-cell patch-clamp recordings to evaluate the functional phenotype of disease-specific and control MSNs. Moreover, gene expression analysis as well as morphometric analysis of MSNs were performed to identify novel treatment targets for DYT-THAP1 dystonia.

## Materials and Methods

### Cultivation of Human iPSC Lines

Both patient-derived DYT-THAP1 iPSC lines (3735-5, 3969-2) were reprogrammed using Sendai viruses and published recently (Baumann et al., 2018). The DYT-THAP1 iPSC line of a family member with reduced penetrance (3737-1) was reprogrammed retrovirally and analyzed within this publication (Table 1). The matched healthy control–derived iPSC lines were characterized previously (Japtok et al., 2015; Glaß et al., 2018). Data of these healthy control lines have also been included in another manuscript (Kutschenko et al., 2021). For the sake of visual clarity, we decided to illustrate bar graphs with pooled data of control cell lines and THAP1 mutation carriers, respectively. For expansion, the iPSCs were cultivated feeder-free in mTeSR medium (Stemcell Technologies, Vancouver, Canada) as colonies and detached using 0.5 mM EDTA for splitting every 4–7 days (WiCell, 2012).

**TABLE 1:** Characteristics of healthy control subjects and DYT-THAP1 dystonia mutation carriers as skin fibroblast donors for iPSC lines used in this study.

**Healthy controls**						
**ID code**		**Gender**		**Age at biopsy**		**Previously published**

Control_1		F		48		Japtok et al., 2015
Control_2		M		34		Glaß et al., 2018

**DYT-THAP1 patients**						

**ID code**	**iPSCs line name**	**Gender**	**Age at biopsy**	**Genotype of locus**	**Previously published**	**Clinical phenotype**

THAP1_1	3735-5	M	77	DYT-THAP1, c.474delA (p.Lys158Asnfs*23)	Baumann et al., 2018	Writer’s dystonia followed by laryngeal dystonia (Zittel et al., 2010)
THAP1_2	3737-1	F	48	DYT-THAP1, c. 474delA (p.Lys158Asnfs*23)	–	Asymptomatic (non-manifesting mutation carrier) (Zittel et al., 2010)
THAP1_3	3969-2	M	38	DYT-THAP1, c.38G > A (p.Arg13His)	Baumann et al., 2018	Generalized dystonia (Zittel et al., 2010)

p.Lys158Asnfs*23 denotes a frame shifting change with lysine at position 158 as the first amino acid, changed into an asparagine and the new termination site 23.

### Differentiation of iPSC Lines Into Striatal Medium Spiny Neurons (MSNs)

The striatal differentiation of iPSCs into MSNs was adapted from two of our previous protocols published by Stanslowsky et al. (2016) and Capetian et al. (2018). First, feeder-free iPSC colonies were detached using 0.5 mM EDTA 3–4 days after splitting. Detached colonies were triturated to smaller pieces, suspended in mTeSR (Stemcell Technologies, Vancouver, Canada), supplemented with 1 μM dorsomorphin (Tocris, Bio-Techne, Minneapolis, United States), 10 μM SB-431542 (Tocris) for neural induction, 1 μM IWP2 as an antagonist of Wnt signaling (Merck, Darmstadt, Germany), and 10 μM Rho kinase (ROCK) inhibitor Y27632 (Stemcell Technologies), and plated on low attachment culture plates to form free-floating embryoid bodies (EBs) at day 0. On day 2, medium was replaced with 1:1 mTeSR/N2 medium (Knockout-DMEM/F-12, with 1:100 N2 supplement; Thermo Fisher Scientific, Waltham, United States) and 1% penicillin, streptomycin, L-glutamine (Thermo Fisher Scientific) supplemented with the same concentrations of dorsomorphin, SB-431542, and IWP2 as mentioned above. From days 4–12, EBs were kept in N2 medium. On day 4, N2 medium was supplemented with dorsomorphin, SB-431542, and IWP2 as well as 0.2 μM purmorphamine (PMA; Enzo Life Sciences, Lörrach, Germany). On days 6 and 8, medium was replaced with N2 medium supplemented with 1 μM IWP2 and 0.2 μM PMA. On day 10, only N2 medium without supplements was used. On day 12, EBs were reduced in size by careful trituration and plated on Matrigel-coated (Corning, United States) six-well plates in N2B27 maturation medium (1:1 DMEM/F-12 and Neurobasal medium containing 1:200 N2, 1:100 B27 without vitamin A; Thermo Fisher Scientific, and 1% penicillin, streptomycin, L-glutamine) with 20 ng/μl brain-derived neurotrophic factor (BDNF; PeproTech, Cranbury, United States), 10 ng/ml glial cell line–derived neurotrophic factor (GDNF; PeproTech) and 50 μM dibutyryl-cAMP (dbcAMP; Sigma-Aldrich, St. Louis, United States). Once the outgrowing cells reached full confluency (days 16–20), they were treated with Accutase (Thermo Fisher Scientific) and replated in smaller aggregates on laminin/poly-DL-ornithine hydrobromide (Thermo Fisher Scientific) coated plates. For terminal differentiation, neuronal cells were replated as single cell suspension after treatment with Accutase (Thermo Fisher Scientific) and cell counting on laminin/poly-DL-ornithine hydrobromide (Thermo Fisher Scientific) coated plates (days 24–30). At each passaging step, 10 μM ROCK inhibitor Y27632 was added to the medium for 48 h. Maturation medium was changed every other day. Fully differentiated neuronal cells were analyzed after 70 days ± 7 days. A total of two to three independent MSN differentiations of each iPSC line (two control and three THAP1 lines, Table 1) was analyzed.

### Immunocytochemistry

Immunofluorescent stainings were performed on day 16 to evaluate outgrowth of neuronal cells from plated EBs, and after 70 ± 7 days to quantify the amount of terminally differentiated MSNs. Cells on coverslips were fixed for 20 min with 4% paraformaldehyde (PFA, Sigma-Aldrich) at room temperature, washed three times with phosphate-buffered saline (PBS, Thermo Fisher Scientific), and incubated for 60 min with blocking buffer (5% goat serum, 1% bovine serum albumin (BSA, Sigma-Aldrich), 0.3% Triton X-100 (Sigma-Aldrich) in PBS). The following primary antibodies were used: rabbit polyclonal anti-GABA **(γ** aminobutyric acid, 1:1,000, Sigma-Aldrich), mouse anti-GABA (1:500, Abcam), rabbit polyclonal anti-TUBB3 (β-tubulin III, 1:1,000, Abcam, Cambridge, United States), mouse IgG2a anti-TUBB3 (1:1,000, Abcam), rabbit polyclonal anti-DARPP32 (dopamine and cAMP-regulated neuronal phosphoprotein 32 kDa, 1:100, Abcam, United States), rat IgG2a anti-CTIP2 (COUP TF1-interacting protein 2, 1:300, Abcam), mouse IgG1 anti-Nestin (1:300, RD systems, Minnesota, United States). Primary antibodies were diluted in blocking buffer and incubated overnight at 4°C. After washing three times with PBS, cells were incubated with the respective secondary antibodies (AlexaFluor 488 or 555, goat anti-mouse, goat anti-rabbit; goat anti-rat; 1:1,000, Thermo Fisher Scientific) for 2 h at room temperature. After two washing steps in PBS, coverslips were mounted using mounting medium supplemented with 0.1% DAPI (4,6-diamidino-2-phenylindole, 10 mg/ml, Thermo Fisher Scientific) for staining of nuclei. To check for background staining, primary antibodies were omitted for one additional coverslip for each staining combination per differentiation. Specimens were visualized with a fluorescence microscope BX61 (Olympus, Shinjuku, Japan) and DP72 camera (Olympus) using the analysis software Cell^*F*^ (Olympus). Four random visual fields per coverslip were taken from one to two independent differentiations for each control and THAP1 line. Cells were counted manually using ImageJ (NIH, Bethesda, United States). Representative images were processed using ImageJ.

### Analysis of Neuronal Morphology and Synaptic Density

Cells were stained with primary antibodies mouse anti-TUBB3 (β-tubulin III, 1:1,000, Abcam) and rabbit anti-GABA (1:1,000, Sigma-Aldrich). Specimens were visualized with fluorescence microscope BX61 (Olympus) and DP72 camera (Olympus) using the analysis software Cell^*F*^ (Olympus). Pictures for analysis were taken at 40-fold magnification and with a constant exposure time for each channel ensuring comparability between images. ImageJ-based plugin NeurphologyJ (Ho et al., 2011) was used to quantify soma, neurites, attachment points, and endpoints as described previously (Stanslowsky et al., 2016). Briefly, the plugin uses two different thresholds for somata and neurites as well as the information of the approximate neurite thickness for detection and quantification of number of somata and neurites, the area of somata and neurites, and the total neurite length within an image. Those parameters were used to normalize neurites by somata to obtain a quantitative expression for total neurite outgrowth within an image. Similarly, the endpoints of a neurite were normalized by soma attachment points to receive an expression for ramification. For quantification of GABAergic synaptic density, the ImageJ plugins SynapCountJ (version 2.0) and NeuronJ (version 1.4.3) (Meijering et al., 2004) were used as described previously (Hensel et al., 2016). In brief, NeuronJ was used to trace a neurite, and this tracing was applied to SynapCountJ, in which GABA-positive spots along the tracing were detected within an intensity threshold. In total, 10–20 images from at least two independent differentiations from two control lines and two THAP lines (THAP1_1, THAP1_3) were investigated.

### Calcium Imaging

To measure intracellular calcium signals, MSNs of three to five independent differentiations (70 ± 7 days) per iPSC line were loaded with the membrane permeable fluorescent dye Fura 2-AM (Sigma-Aldrich). The MSNs were incubated for 20 min at 37°C with Fura 2-AM in a standard bath solution (containing 140 mM NaCl, 5 mM KCl, 2 mM CaCl_2_, 10 mM glucose, and 10 mM HEPES, adjusted to pH 7.4 with NaOH). To image intracellular calcium, Fura 2-AM loaded MSNs were excited at wavelengths of 340/380 nm and monitored every 300 ms at 510 nm. Recordings were performed on an upright microscope Axioskop 2 FS plus (Carl Zeiss MicroImaging GmbH, Göttingen, Germany) connected to a Till Vision Imaging System (TILL Photonics, Gräfelfing, Germany). Emitted fluorescence was collected by a charge-coupled device (CCD) camera as described previously (Stanslowsky et al., 2014, 2016). To monitor spontaneous intracellular Ca^2+^ changes, MSNs with a preferably multipolar dendritic morphology were imaged for 6 min. Further, the neurotransmitters glycine (100 μM), GABA (100 μM), acetylcholine (100 μM), and glutamate (50 μM) were applied to MSNs after 1 min baseline condition, and the provoked intracellular Ca^2+^ changes were recorded for 1 min followed by perfusion with the bath solution. Note, glycine- and GABA-induced Ca^2+^ peaks suggest a slight depolarizing effect of these neurotransmitters in some cells, most likely due to a high intracellular chloride concentration (Wegner et al., 2008). The amplitudes of Ca^2+^ signals after neurotransmitter application were measured, and the percentage of responsive cells was calculated from each recording. Recordings were terminated by application of KCl (50 mM) to induce neuronal depolarization and ensure the viability and excitability of the recorded cells. After background subtraction, the 340/380 nm excitation ratio for Fura 2-AM was calculated, which increases as a function of the cytosolic free Ca^2+^ concentration ([Ca^2+^]_*i*_). For analysis of spontaneous Ca^2+^ transients, only Ca^2+^ signals with a 340/380 nm excitation ratio of Fura 2-AM of ≥ 0.02 of individual MSNs were used. For analysis of response amplitudes after neurotransmitter application, only Ca^2+^ signals with a 340/380 nm excitation ratio of Fura 2-AM ≥ 0.05 of individual MSNs were used. For analysis, the peak amplitude of Ca^2+^ transients was used. Per independent differentiation, at least two coverslips with 10–40 MSNs from two control lines and three THAP1 lines were investigated. Data from at least three independent differentiations for each control and THAP1 line were analyzed.

### Quantitative Real-Time PCR

For RNA extraction, iPSCs and differentiated MSNs (collected at 70 ± 7 days) were processed using RNeasy Mini Kit (Qiagen, Venlo, Netherlands), including a column DNase digestion. The quality of total RNA was checked by NanoDrop analysis (Nanodrop Technologies, Wilmington, Delaware, United States). A total of 250 ng of RNA was transcribed into cDNA using QuantiTect reverse transcription kit (Qiagen). Quantitative real-time PCR reaction analysis was performed using Power SYBR PCR Green Master Mix (Thermo Fisher Scientific) in a StepOnePlus cycler (Thermo Fisher Scientific) under following conditions: 95°C for 10 min followed by 40 cycles of 95°C for 15 s and 60°C for 1 min. Per reaction, a total amount of 1.75 ng RNA transcribed to cDNA was analyzed and 1.75 μM of forward and reverse primers were used. As recommended by the MIQE-guidelines (Bustin et al., 2009), specificity of PCR products was ensured by melting curve analysis, and equal PCR efficiency of all primer pairs was validated by serial dilutions of cDNA. For the following primer pairs and PCR products, the PCR efficiency or detection limit Ct < 30 were not meeting the quality criteria by the MIQE guidelines and, therefore, not included in further analysis: primer pairs for target genes *PVALB* encoding parvalbumin. The threshold cycle (Ct) values of target genes were normalized to expression of endogenous references beta2-microglobulin (B2M), glyceraldehyde 3-phosphate dehydrogenase (GAPDH) and β-actin with the following formula: [Ct (target) − Ct (reference) = ΔCt]. Gene expression of two independent differentiations per cell line (two control lines and two THAP1 lines (THAP1_2, THAP1_3) and each in triplicate were used for analysis of the target gene *SST* encoding somatostatin and illustrated as means ± standard error of the mean (SEM). Gene expression of three independent differentiations per cell line (two control lines and two THAP1 lines (THAP1_2, THAP1_3) and each in triplicate were used for analysis of target genes *GAD67, FOXP1, CTIP2, TUBB3, MAP2* and illustrated as means ± SEM. For a list of all primer sequences, including GABA_*A*_ receptor subunits and voltage-gated calcium channels see Supplementary Tables 1–3.

### Electrophysiology

Electrophysiological patch-clamp measurements of MSNs were performed after 70 ± 7 days of differentiation *in vitro* from at least three independent differentiations per iPSC line as described previously (Stanslowsky et al., 2014, 2016). To identify MSNs, only medium-sized multipolar neurons were used for analysis. This morphological selection approach previously yielded > 90% of DARPP-32 positive MSNs in whole-cell recordings coupled to biocytin filling (Capetian et al., 2018). Patch-clamp pipettes with final resistances of 3–4 MΩ were filled with an internal solution (153 mM KCl, 1 mM MgCl_2_, 10 mM HEPES, and 5 mM EGTA, adjusted to pH 7.3 with KOH, 305 mOsm). The external bath solution contained 142 mM NaCl, 8 mM KCl, 1 mM CaCl_2_, 6 mM MgCl_2_, 10 mM glucose, and 10 mM HEPES, adjusted to pH 7.4 with NaOH, 325 mOsm. The combination of external and internal solutions produced a chloride equilibrium potential near 0 mV. Whole-cell currents were low-pass filtered at 2.9 kHz, digitized at 10 kHz using an EPC-10 amplifier (HEKA Elektronik, Harvard Bioscience, Massachusetts, United States), and analyzed with PatchMaster and FitMaster software (HEKA). Sodium and potassium ion currents were elicited by depolarizing voltage steps in increments of 10 mV from a holding potential of −70 to 40 mV. Miniature postsynaptic currents (mPSCs), indicating mostly GABAergic synaptic activity in MSNs (Stanslowsky et al., 2016; Capetian et al., 2018) were acquired at a holding potential of −70 mV in voltage-clamp mode. For quantitative analysis only mPSC amplitudes between 10 and 100 pA were measured to exclude noise artifacts (<10 pA) and action potential activity (>100 pA) of the recorded neuron. For advanced analysis of inhibitory mPSCs in future MSN studies, the use of TTX (tetrodotoxin) in the extracellular solution as well as N-ethyl lidocaine and CsCl in the pipette solution is an important approach to record the precise frequency and amplitude of iPSCs under voltage-clamp configuration. Spontaneous and evoked action potentials (APs) were recorded in current-clamp mode at holding potentials of −50 to −70 mV. For recordings, data from two control lines and two THAP1 lines (THAP1_2, THAP1_3) from at least two independent differentiations were investigated.

### Statistical Analysis

Statistical analysis was performed using GraphPad Prism 5 Software (GraphPad Software, San Diego, California, United States). All data of MSNs from healthy controls and THAP1 lines were pooled in control and THAP1 groups for the respective experiments and are presented as mean ± standard error of the mean (SEM). When the two groups showed normal distribution, a two-tailed unpaired *t*-test, in other cases a non-parametric Mann-Whitney test was calculated comparing the two groups (control versus THAP1). A one-way ANOVA or two-way ANOVA followed by Bonferroni *post hoc* test was used for multiple comparisons when comparing more than two groups. The significance level (*p*-value) was set to *p* < 0.05 with ^∗^*p* < 0.05, ^∗∗^ *p* < 0.01, ^∗∗∗^ *p* < 0.001.

## Results

### Differentiation of iPSCs Into Medium Spiny Neurons (MSNs)

**FIGURE 1:**
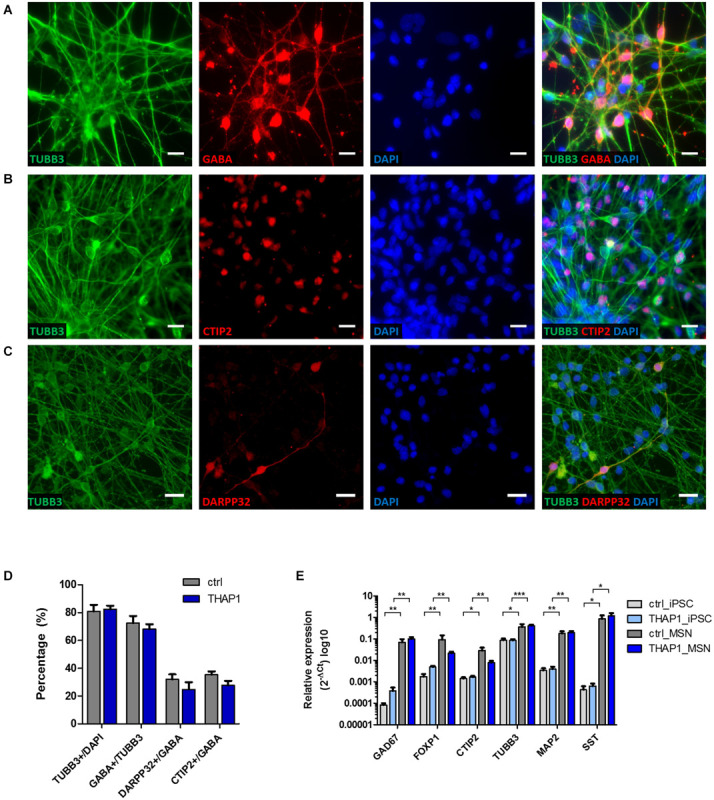
Differentiation of iPSCs derived from DYT-THAP1 mutation carriers and healthy controls into striatal medium spiny neurons (MSNs). **(A–C)** Mature MSNs at day 70 (±7 days) of differentiation expressed the neuronal markers β-tubulin III (TUBB3), γ-aminobutyric acid (GABA) and MSN-specific striatal markers dopamine- and cAMP-regulated neuronal phosphoprotein 32kDa (DARPP32) and COUP TF1-interacting protein 2 (CTIP2) as shown by representative images of immunofluorescent stainings. Nuclei were stained with 4,6-diamidino-2-phenylindole (DAPI). Scale bar indicates 20 μm. **(D)** About 80% of DAPI-stained MSNs expressed the neuronal marker TUBB3, of which the majority were GABA-positive cells. About 30% of GABA-positive cells co-expressed DARPP32 and CTIP2 showing terminal differentiation into striatal MSNs. Quantitative estimation of neuronal and MSN-specific markers was similar for THAP1 and control MSNs. Data are presented as means ± SEM from one to two independent differentiations for each control and THAP1 line. **(E)** Expression analysis of iPSCs and MSNs derived from DYT-THAP1 patients and healthy controls by quantitative real-time PCR. Compared with iPSCs, THAP1 and control MSNs expressed significantly elevated levels of markers for neuronal origin (FOXP1, forkhead box protein P1; TUBB3, β-tubulin III; MAP2, microtubule-associated protein 2), GABAergic origin (GAD67, glutamic acid decarboxylase) and MSN-specific striatal origin (CTIP2, COUP TF1-interacting protein 2). Expression of neuronal, GABAergic and MSN-specific markers was similar for THAP1 and control MSNs. Data are presented as means ± SEM in logarithmic scale (log_10_) from at least two independent differentiations for control and THAP1 lines (*p < 0.05, **p < 0.01, ***p < 0.001, parametric *t*-test or non-parametric Mann-Whitney test).

After 70 days of iPSC differentiation, we generated striatal medium spiny neurons (MSNs) as main neuronal cell type for modeling dystonia *in vitro* (Figure 1). After plating EBs, neural progenitor cells expressed Nestin around day 18 during maturation (data not shown). The quantitative immunocytochemical analysis of mature MSNs revealed 80% β-tubulin III (TUBB3)-positive neurons of which ∼68% were positive for the neurotransmitter γ-aminobutyric acid (GABA). Approximately ∼30% of GABAergic MSNs co-expressed the striatal markers cAMP-regulated neuronal phosphoprotein 32 kDa (DARPP32) and COUP TF1-interacting protein 2 (CTIP2) (Figures 1A–D). There was no significant difference between healthy control and THAP1 MSNs. The mRNA expression of neuronal and striatal markers using quantitative real-time PCR showed a significant upregulation compared to the iPSC origin but was not significantly different for THAP1 and control MSNs (Figure 1E). The upregulation of glutamic acid decarboxylase (GAD67), transcription factor forkhead box protein P1 (FOXP1), that is associated with MSN maturation (Precious et al., 2016), and the mature neuronal marker microtubule-associated protein 2 (MAP2) confirmed the neuronal and mainly GABAergic phenotype of MSNs underlining the results from the immunostainings. The difference in the expression of FOXP1 and CTIP2 is not statistically significant for both MSN groups.

In addition, the genomic expression of somatostatin (SST) indicates the presence of striatal GABAergic interneurons (Figure 1E).

### Morphological Analysis and Synaptic Density of MSNs

#### Normal Neuronal Morphology and Synaptic Density in THAP1 MSNs

**FIGURE 2:**
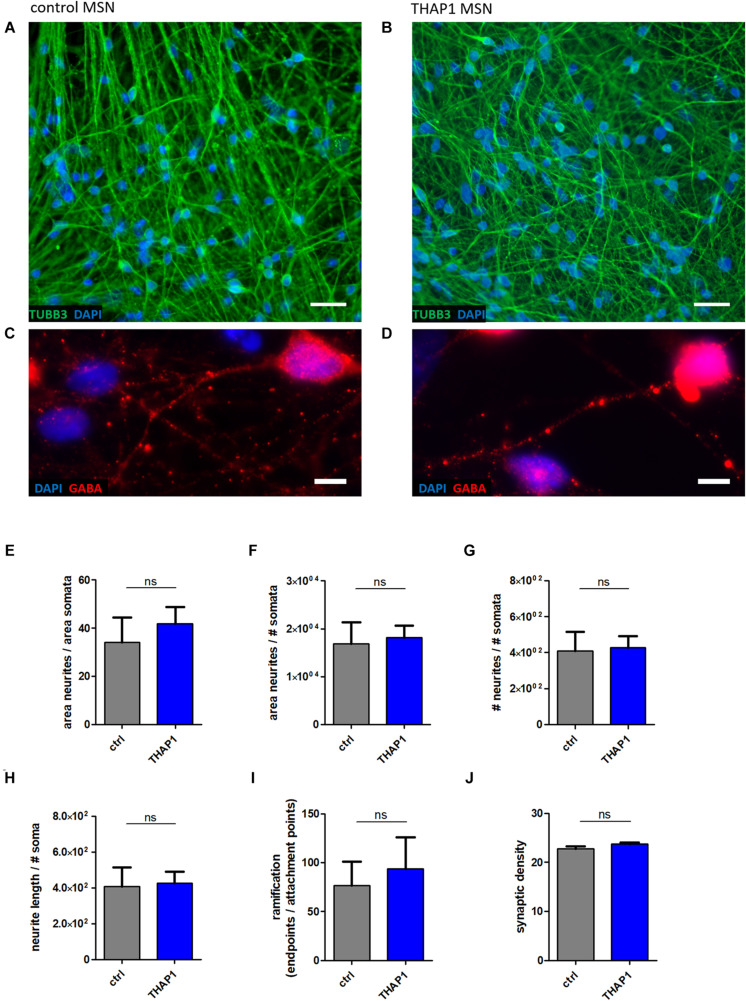
Neuronal morphology and GABAergic synaptic density of MSNs from DYT-THAP1 patients and healthy controls. Control **(A,C)** and THAP1 MSNs **(B,D)** were stained for neuronal marker β-tubulin III (TUBB3) and for γ-aminobutyric acid (GABA) to analyze neuronal morphology using ImageJ plugin Neurphology and to quantify GABAergic synapses using ImageJ plugins SynapCountJ and NeuronJ. Nuclei were stained with 4,6-diamidino-2-phenylindole (DAPI). Scale bar indicates 20 μm **(A,B)** and 5 μm **(C,D)**. Total neurite outgrowth parameters were quantified by normalization of neurites by somata. **(E)** The area of neurites was divided by the area of somata or **(F)** the number of somata (# somata). **(G)** The number of neurites (# neurites) was divided by the number of somata (# somata). **(H)** Total neurite length was obtained by neurite length divided by # somata. **(I)** For the evaluation of total ramification, the number of endpoints of neurites was normalized by the number of attachment points at the soma. All of these neurite parameters **(E–I)** were comparable between THAP1 and control MSNs. **(J)** No significant difference of THAP1 and control MSNs was detected in regard to the amount of GABAergic synapses. Abbreviation: ns, not significant. Data are presented as means ± SEM from two to three independent differentiations for THAP1 and control lines (non-parametric Mann-Whitney test).

The morphological analysis of MSN neurites (using β-tubulin III (TUBB3) staining, Figures 2A,B) displayed no differences in total neurite length as detected by analysis of parameters, including neurite length, neurite area, and number of neuronal somata per acquired image (Figures 2E–H). In addition, a similar degree of ramification between THAP1 and control MSNs was detected by analyzing the number of attachment points of neurites at soma and endpoints of neurites (Figure 2I). The quantification of GABA-positive boutons in MSNs for the evaluation of synapse formation revealed a similar synaptic density between THAP1 and control MSNs (Figures 2C,D,J).

### Ca^2+^ Signaling in MSNs

#### Elevated Basal Intracellular Ca^2+^ Levels in THAP1 MSNs

**FIGURE 3:**
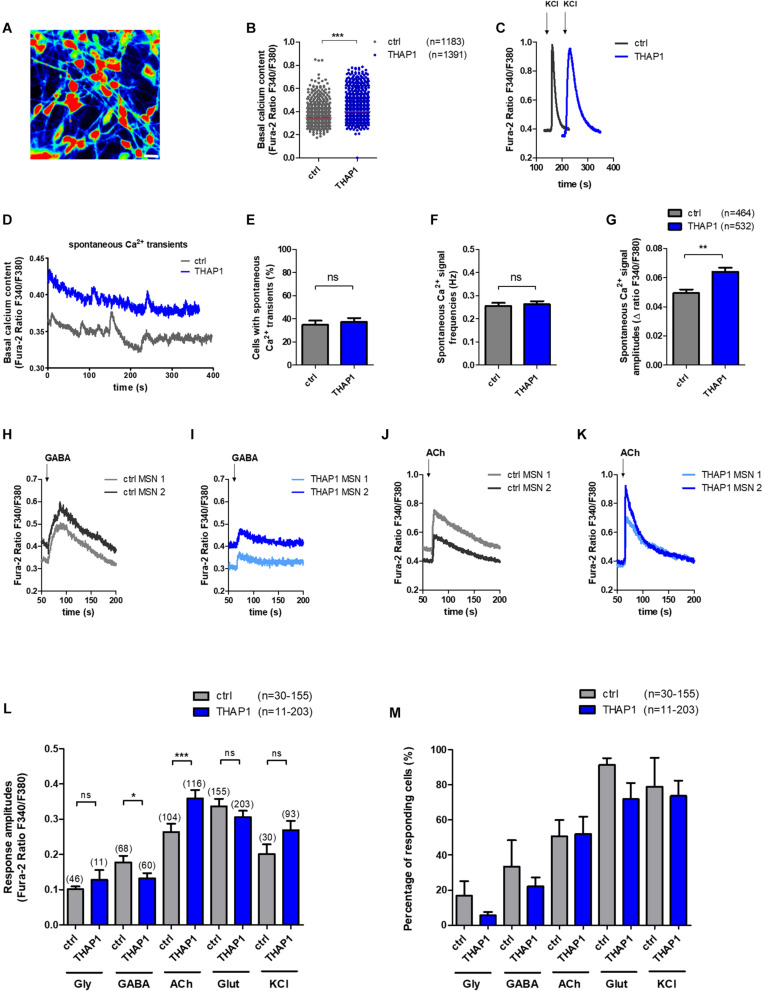
Spontaneous intracellular calcium (Ca^2+^) dynamics and neurotransmitter-induced Ca^2+^ signaling of MSNs derived from DYT-THAP1 patients and healthy controls. Intracellular Ca^2+^ transients are presented as ratios of the fluorescence signals obtained at 340 and 380 nm (F_340_/F_380_). **(A)** Representative image of Ca^2+^ recording of THAP1 MSNs loaded with Fura-2. Scale bar indicates 30 μm. **(B)** Basal intracellular Ca^2+^ levels were significantly elevated in THAP1 (*n* = 1,391) compared with control MSNs (*n* = 1,183, ***p < 0.001, non-parametric Mann-Whitney test). **(C)** Representative traces of intracellular Ca^2+^ changes of control and THAP MSNs induced by application of KCl (50 mM) to monitor the viability and excitability of MSNs. **(D)** Representative traces of spontaneous Ca^2+^ peaks of control and THAP MSNs. **(E)** The percentage of cells showing spontaneous Ca^2+^ transients were not significantly different between both MSN groups (∼35%, control *n* = 464, THAP1 *n* = 532, unpaired *t*-test). **(F)** Spontaneous Ca^2+^ signal frequencies of MSNs were not significantly different between MSNs of both groups (∼0.26 Hz, control *n* = 464, THAP1 *n* = 532, non-parametric Mann-Whitney test). **(G)** Spontaneous Ca^2+^ amplitudes of MSNs were significantly elevated in THAP1 MSNs (*n* = 532) compared with control MSNs (*n* = 464, **p < 0.01, non-parametric Mann-Whitney test). **(H,I)** Representative traces of intracellular Ca^2+^ changes of two Fura-2 loaded control and THAP1 neurons induced by separate application of GABA (100 μM) and **(J,K)** acetylcholine (ACh, 100 μM). **(L)** Cytosolic Ca^2+^ response amplitudes upon separate applications of KCl and the neurotransmitters glycine (Gly, 100 μM), γ-aminobutyric acid (GABA, 100 μM), acetylcholine (ACh, 100 μM) and glutamate (Glut, 50 μM) normalized to the basal Ca^2+^ level of MSNs (*n* = number of cells) showed significantly lower Ca^2+^ peaks upon application of GABA and elevated Ca^2+^ peaks upon application of acetylcholine in THAP1 compared with control MSNs (control *n* = 30–155, THAP1 *n* = 11–203, *p < 0.05, ***p < 0.001, non-parametric Mann-Whitney test). **(M)** Percentage of cells responding to neurotransmitter application and KCl with Ca^2+^ rises obtained from experiments shown in subfigure L was similar for THAP1 and control MSNs (control *n* = 30–155, THAP1 *n* = 11–203, *p* > 0.05, non-parametric Mann-Whitney test). Abbreviation: ns, not significant. Data from at least three independent differentiations for each control and THAP1 line were analyzed. Data are presented as means ± SEM.

A representative image of intracellular Ca^2+^ recordings of Fura-2 loaded THAP1 MSNs is shown in Figure 3A. THAP1 MSNs showed significantly elevated basal intracellular Ca^2+^ levels compared with controls (THAP1 0.400 ± 0.001, control 0.347 ± 0.001, *p* < 0.001, Figure 3B). The viability and excitability of MSNs was monitored by application of KCl and was similar for control and THAP1 MSNs; a representative image is shown in Figure 3C. Furthermore, a representative image of spontaneous Ca^2+^ transients of MSNs is shown in Figure 3D. The percentage of MSNs with spontaneous Ca^2+^ currents was not significantly different between THAP1 (37.3 ± 3.4%) and control MSNs (34.8 ± 3.6%) (Figure 3E). In addition, the frequency of spontaneous Ca^2+^ currents was similar for MSNs in both groups (THAP1 0.263 ± 0.013 Hz, control 0.255 ± 0.014 Hz, Figure 3F). However, the amplitudes of spontaneous Ca^2+^ transients were significantly elevated in THAP1 compared with control MSNs (THAP1 0.064 ± 0.003, control 0.049 ± 0.003, *p* = 0.002, Figure 3G).

#### Lower Ca^2+^ Amplitudes in Response to GABA and Elevated Peaks Upon Acetylcholine Application in THAP1 MSNs

Representative traces of intracellular Ca^2+^ transients of Fura-2 loaded control and THAP1 MSNs induced by separate application of GABA (100 μM) and acetylcholine (100 μM) are shown in Figures 3H–K. Analysis of neurotransmitter-induced Ca^2+^ signaling revealed that THAP1 MSNs showed significantly lower amplitudes of Ca^2+^ transients in response to application of GABA (THAP1 0.06 ± 0.006, control 0.11 ± 0.013, *p* < 0.05) and significantly elevated amplitudes in response to acetylcholine (THAP1 0.36 ± 0.024, control 0.26 ± 0.023, *p* < 0.001) although there were no differences after applications of glycine and glutamate as well as KCl (Figure 3L). The percentage of cells responding with Ca^2+^ rises to individual neurotransmitter applications were similar for both MSN groups (Figure 3M) including the ∼20–30% of MSNs with small depolarizing GABA effects (Stanslowsky et al., 2016).

### Expression of GABA_*A*_ Receptor Subunits and Voltage-Gated Ca^2+^ Channels

#### Altered Expression of GABA_*A*_ Receptor Subunits in THAP1 MSNs

**FIGURE 4:**
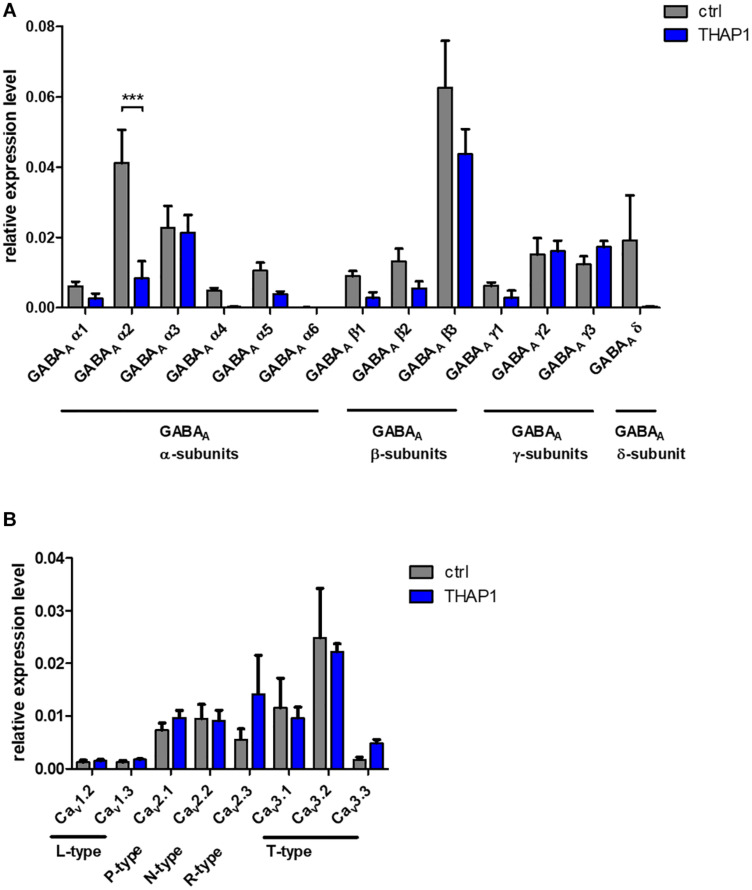
Expression analysis of ion channels in MSNs from DYT-THAP1 patients and healthy controls by quantitative real-time PCR. **(A)** Genomic expression of γ-aminobutyric acid type A (GABA_*A*_) receptor subunits α(1–6), β(1–3), γ(1–3) and δ showed a significantly lower expression level of GABA_*A*_ α2 subunit in THAP1 compared with control MSNs. **(B)** Genomic expression of voltage-gated Ca^2+^-channels was similar in THAP1 and control MSNs. Data are presented as means ± SEM from three independent differentiations for each control and THAP1 line (***p < 0.001, two-way ANOVA with Bonferroni *post hoc* test).

The expression of genes encoding GABA_*A*_ receptor subunits α, β, and δ tended to be reduced in THAP1 compared with control MSNs (Figure 4A), whereas γ-subunits were expressed similarly. A significantly lower expression level was detected for the gene encoding the α2-subunit (*p* < 0.001), and α4 and the extrasynaptic δ-subunit were also markedly downregulated in THAP1 MSNs.

#### Similar Expression of Voltage-Gated Ca^2+^ Channel Subtypes in MSNs

Genomic expression levels of voltage-gated Ca^2+^ channel subtypes were similar in THAP1 and control MSNs (Figure 4B). Therefore, these channels might not contribute significantly to the elevated basal Ca^2+^ concentrations in THAP1 neurons.

### Properties of Action Potentials (APs) and Synaptic Activity in MSNs

Electrophysiological recordings from iPSC-derived MSNs of THAP1 patients and healthy controls were performed to assess passive membrane properties, voltage-gated ion channels, action potentials and synaptic functionality (Table 2).

**TABLE 2:** Electrophysiological properties recorded by whole-cell patch-clamp of iPSC-derived MSNs from two DYT-THAP1 mutation carriers (THAP1_2 and THAP1_3) and two healthy controls.

	**Control**			**DYT-THAP1**		
**Functional properties**	**Control_1 (*n* = 21)**	**Control_2 (*n* = 14)**	**Control (*n* = 35)**	**THAP1_2 (*n* = 39)**	**THAP1_3 (*n* = 13)**	**THAP1 (*n* = 52)**
I_*Na*_ max. amplitudes (pA/pF)	−106.2 ± 18.8	−126.6 ± 19.5	−116.4 ± 10.2	−109.7 ± 16.2	−136.6 ± 45.0	−123.2 ± 13.5
I_*K*_ max. amplitudes (pA/pF)	148.4 ± 30.2	125.3 ± 26.3	136.9 ± 11.5	147.3 ± 18.0	140.0 ± 28.8	143.7 ± 3.7
Resting membrane potential (mV)	−39.1 ± 2.2	−23.3 ± 3.1	−32.9 ± 2.2	−42.30 ± 2.0	−37.9 ± 1.5	−41.2 ± 1.5**
Membrane capacitance (pF)	30.5 ± 3.9	17.8 ± 3.1	25.4 ± 2.8	21.1 ± 2.1	21.7 ± 6.0	21.2 ± 2.1
Input resistance (MOhm)	996.3 ± 275.2	553.9 ± 195.2	819.4 ± 184.2	566.6 ± 167.4	337.3 ± 60.22	510.4 ± 127.5
Cells with single evoked APs (%)	48.9 ± 24.8	87.5 ± 12.5	68.2 ± 15.1	19.2 ± 12.7	45.8 ± 20.8	31.0 ± 11.8
Cells w. repetitive evoked APs (%)	51.1 ± 24.8	12.5 ± 12.5	31.8 ± 15.1	80.8 ± 12.7	54.2 ± 20.8	69.0 ± 11.88
Amplitude (mV) of evoked APs	56.9 ± 5.1	57.8 ± 5.0	57.2 ± 3.7	73.0 ± 2.9	58.9 ± 5.2	68.8 ± 2.7*
Duration (ms) of evoked APs	3.0 ± 0.4	3.5 ± 0.7	3.2 ± 0.4	3.5 ± 0.4	2.9 ± 0.4	3.3 ± 0.3
AHP amplitude (mV)	7.6 ± 1.9	3.2 ± 0.7	6.0 ± 1.3	7.1 ± 1.0	2.1 ± 0.2	5.6 ± 0.8
Time to peak AHP (ms)	21.6 ± 4.0	19.5 ± 3.0	20.9 ± 2.7	44.1 ± 7.9	30.3 ± 6.5	39.9 ± 5.9**
Cells with spontaneous APs (%)	58.5 ± 20.3	56.3 ± 6.3	57.6 ± 11.3	78.1 ± 7.0	89.6 ± 10.4	83.2 ± 6.0
Frequency of spontaneous APs (Hz)	0.50 ± 0.10	0.47 ± 0.10	0.49 ± 0.07	1.22 ± 0.21	0.74 ± 0.20	1.07 ± 0.16*
Amplitude of spontaneous APs (mV)	49.0 ± 3.1	42.7 ± 6.7	46.5 ± 3.2	42.3 ± 4.2	33.6 ± 4.1	39.7 ± 3.3
Cells with miniature PSCs (%)	96.3	100.0	98.2	87.0	95.8	90.5
Miniature PSC frequencies (Hz)	3.2 ± 0.71	2.4 ± 0.63	2.9 ± 0.48	1.1 ± 0.42	1.5 ± 0.50	1.2 ± 0.33***
Miniature PSC amplitudes (pA)	22.6 ± 2.2	21.0 ± 3.1	21.9 ± 1.8	23.0 ± 2.5	21.9 ± 2.7	22.7 ± 1.9

*I*_*Na*_, Voltage-gated sodium; I_*K*,_ potassium currents; APs, action potentials; AHP, afterhyperpolarization; PSC, postsynaptic currents. Data are given as means ± SEM (*p < 0.05, **p < 0.01, ***p < 0.001, non-parametric Mann-Whitney test). Bold values mean significant results.

**FIGURE 5:**
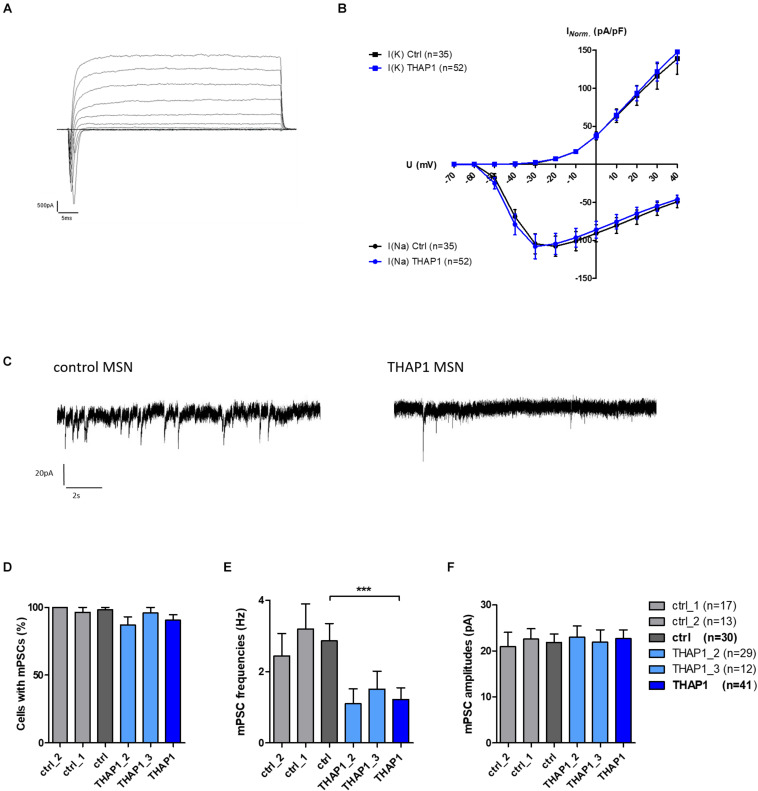
Voltage-gated ion currents and spontaneous synaptic activity of MSNs derived from DYT-THAP1 patients and healthy controls. **(A)** Voltage-gated sodium inward and potassium outward currents of THAP1 MSN at 70 days *in vitro* were recorded in whole-cell voltage-clamp mode by depolarizing steps in increments of 10 mV from a holding potential of –70 to 40 mV. **(B)** Ion currents normalized for individual cell sizes based on the capacitance of cell membrane (pA/pF) were not significantly different between THAP1 (*n* = 52) and control MSNs (*n* = 35, two-way ANOVA with Bonferroni *post hoc* test). **(C)** Traces of miniature postsynaptic currents (mPSC), indicating spontaneous synaptic events from MSN afferents, were recorded in whole-cell voltage-clamp mode at a holding potential of –70 mV. **(D)** Percentage of cells with mPSCs was similar for THAP1 and control MSNs (non-parametric Mann-Whitney test). **(E)** Interestingly, mPSC frequencies were significantly lower for THAP1 (*n* = 41) compared with control MSNs (*n* = 30, ***p < 0.001, non-parametric Mann-Whitney test). **(F)** The mPSC amplitudes were not markedly different between THAP1 and control MSNs (non-parametric Mann-Whitney test). Data from two control lines and two THAP1 lines (THAP1_2, THAP1_3) from at least two independent differentiations were analyzed. Data are presented as means ± SEM.

Neurons from the THAP1 (*n* = 52) and control (*n* = 35) groups showed sodium inward and potassium outward currents upon depolarizing steps in increments of 10 mV from a holding potential of −70 to 40 mV (Figure 5A). After normalization of ion current amplitudes for individual cell sizes based on the capacitance of cell membrane (pA/pF), both groups did not significantly differ (Figure 5B). However, resting membrane potentials were significantly more negative in THAP1 MSNs, and capacitances of the cell membrane and input resistances did not show group differences (Table 2).

#### Lower mPSC Frequencies in THAP1 MSNs

The percentage of cells with miniature postsynaptic currents (mPSCs), indicating spontaneous synaptic events from MSN afferents, was slightly lower in THAP1 (91%) compared with control MSNs (98%) (Figures 5C,D). The mPSC amplitudes were similar in both groups, but the mPSC frequencies were significantly lower in THAP1 (1.2 ± 0.33 Hz) than in control MSNs (2.9 ± 0.48 Hz) (*p* < 0.001, Figures 5E,F). In addition, MSNs derived from the asymptomatic carrier exhibited similar action potential properties and synaptic activity in relation to mPSCs in respect to healthy control lines and MSNs derived from a symptomatic carrier.

#### Elevated Amplitude and Frequency of APs in THAP1 MSNs

**FIGURE 6:**
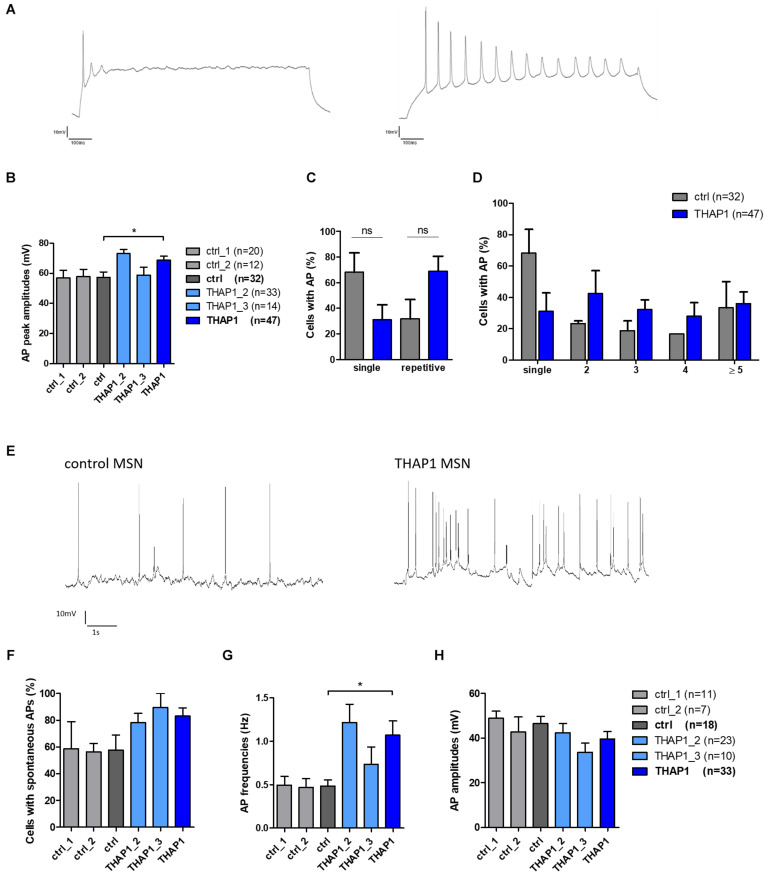
Evoked and spontaneous action potentials (APs) of MSNs from DYT-THAP1 patients and healthy controls. **(A)** Example of THAP1 MSNs firing single and repetitive action potentials upon depolarization in current-clamp mode from a holding potential of approximately –70 mV. **(B)** Action potential peak amplitudes were significantly elevated in THAP1 (*n* = 47) compared with control MSNs (*n* = 32, *p < 0.05, unpaired *t*-test). **(C)** Percentages of neurons with single or repetitive action potentials were not significantly different between MSNs of both groups (non-parametric Mann-Whitney test). **(D)** Percentages of neurons with single or repetitive (2, 3, 4, ≥ 5) action potentials were not significantly different between MSNs of both groups although overall there was a tendency to more THAP1 neurons with repetitive APs (two-way ANOVA with Bonferroni *post hoc* test). **(E)** Spontaneous AP firing was measured at a holding potential of approximately –60 mV showing more APs in THAP1 than control MSN. **(F)** Percentage of cells with spontaneous APs was comparable for THAP1 and control MSNs. **(G)** Interestingly, frequencies of spontaneous APs were significantly elevated for THAP1 (*n* = 33) compared with control MSNs (*n* = 18, *p < 0.05, non-parametric Mann-Whitney test). **(H)** Amplitudes of spontaneous APs were similar for THAP1 and control MSNs. Abbreviation: ns, not significant. Data from two control lines and two THAP1 lines (THAP1_2, THAP1_3) from at least two independent differentiations were analyzed. Data are presented as means ± SEM.

Single action potentials (APs) evoked by depolarizing current injections from a holding potential of approximately −70 mV were fired from the majority of control MSNs (68%) compared with only 32% in the THAP1 group (Table 2). Repetitive evoked spiking was more often recorded for THAP1 (69%) than control MSNs (31%) but without reaching a significant difference (Table 2 and Figures 6C,D). The amplitudes of evoked action potentials were significantly (*p* = 0.011) elevated for THAP1 (68.8 ± 2.7 mV) compared with control MSNs (57.2 ± 3.7 mV) (Figures 6A,B), and AP duration was similar in both groups (THAP1 3.3 ± 0.3 ms, control 3.2 ± 0.4 ms). Although the amplitude of afterhyperpolarization (AHP) was comparable in both groups (THAP1 5.6 ± 0.8 mV, control 6.0 ± 1.3 mV), the time to peak AHP was significantly (*p* = 0.005) longer in THAP1 MSNs (THAP1 39.9 ± 5.9 ms, control 20.9 ± 2.7 ms, Table 2). Spontaneous APs were recorded for 83% THAP1 and 58% control MSNs, which was not significantly different (Figures 6E,F). Whereas the frequency of spontaneous APs was significantly elevated in THAP1 (1.07 ± 0.16 Hz) compared with control MSNs (0.49 ± 0.07 Hz), the amplitude of APs was comparable for both groups (THAP1 39.7 ± 3.3 mV, control 46.5 ± 3.2 mV) (Figures 6G,H). Differences in repetitive firing pattern and resting membrane potential of MSNs could influence the differential expression of not only GABA receptor subunits, but also potassium channels.

## Discussion

For modeling neurodegenerative diseases and movement disorders, our protocols for differentiation of iPSCs toward striatal neurons have been successfully used previously (Stanslowsky et al., 2016; Capetian et al., 2018; Glaß et al., 2018). Also, it seems probable that a minor proportion of differentiated cells did not completely mature to MSNs as we recorded some GABA-evoked intracellular calcium signals. We adapted those established *in vitro* protocols to generate iPSC-derived striatal MSNs from DYT-THAP1 dystonia patients and healthy controls to gain better insights into the pathophysiological processes of this genetic dystonia. Previous studies using iPSC-derived neurons from *THAP1* mutation carriers showed higher *THAP1* levels in these neurons and autoregulation of THAP1 gene expression due to binding to its own promotor (Erogullari et al., 2014) as well as transcriptional alterations of the cortical network (Baumann et al., 2021). However, the molecular and functional phenotype of iPSC-derived THAP1 MSNs has not been investigated so far.

After 70 days of differentiation, MSNs expressed not only the neuronal markers FOXP1, TUBB3, MAP2, and GAD67, but also the MSN-specific markers DARPP32 and CTIP2. As shown by immunocytochemistry and expression-level analysis using quantitative PCR, the iPSC-derived striatal neuronal properties regarding these markers were similar for all control and THAP1 lines arguing against a developmental phenotype. A portion of differentiated neuronal cells exhibited molecular characteristics of striatal interneurons expressing somatostatin (Muñoz-Manchado et al., 2018; Tepper et al., 2018).

Interestingly, differences were found on the expression level of GABA_*A*_ receptor subunits: the gene encoding for GABA_*A*_ α2 subunit was significantly downregulated in THAP1 compared with control MSNs. It is suggested that the GABA_*A*_ α2 subunit is specific for MSNs in the mouse striatum (Boccalaro et al., 2019). This subunit is also likely to be important for other projection neurons, including neurons in the hippocampus of α1-subunit-null mice (Schneider Gasser et al., 2007). On the other hand, this α2 subunit seems to play only a minor role in interneurons (Pirker et al., 2000; Schneider Gasser et al., 2007; Boccalaro et al., 2019).

Due to downregulation of the gene encoding GABA_*A*_ receptor α2 subunit in our striatal THAP1 MSNs, a GABAergic disinhibition as potential pathophysiological mechanism of DYT-THAP1 may be assumed. Correspondingly, it was suggested in the literature that a loss of afferent inhibition onto MSNs can result in dystonic symptoms (Gittis and Kreitzer, 2012; Bode et al., 2017; Balint et al., 2018).

In line with this hypothesis, a markedly lower calcium response of THAP1 MSNs to GABA was displayed using calcium imaging in our study. Because the synaptic afferents of striatal MSNs are mainly GABAergic (Golas, 2018; Boccalaro et al., 2019), the transmission via GABA_*A*_ receptors is crucial for neuronal function. However, during differentiation of human fetal midbrain-derived neural progenitors, GABA could have a slight depolarizing effect in some cells, presumably due to chloride efflux driven by a high intracellular chloride concentration (Wegner et al., 2008; Schaarschmidt et al., 2009). In calcium imaging, a minority of control and THAP1 MSNs showed a small GABA-induced calcium signal suggesting a depolarization and indirect activation of voltage-gated calcium channels. Because GABA_*A*_ receptor–mediated effects switch from depolarization to hyperpolarization during neonatal brain development (Obrietan and van den Pol, 1995; van den Pol et al., 1998; Saint-Amant and Drapeau, 2000; Ben-Ari, 2002; Takayama and Inoue, 2004), we assume that these differential GABA actions are also present during maturation of striatal MSNs. In human iPSC-derived bioengineered neuronal organoids, the developmental GABA polarity switch was observed after day 40, indicating progressive neuronal network maturation (Zafeiriou et al., 2020).

Moreover, we revealed that THAP1 MSNs had elevated basal intracellular calcium levels. It was shown that intracellular calcium dynamics play an important role in the dysregulation of neurons in movement disorders, such as Parkinson’s disease, Huntington disease, and other types of dystonia (Jinnah et al., 2000; Iwabuchi et al., 2013; Schöndorf et al., 2014; HD iPSC Consortium, 2017; Helassa et al., 2017; Raymond, 2017; Capetian et al., 2018).

However, we found not only elevated basal intracellular calcium, but also increased calcium amplitudes after application of acetylcholine in THAP1 compared with control MSNs. It is known from the literature that changes in the cholinergic system play an important pathophysiological role in various forms of dystonia (Breakefield et al., 2008; Bonsi et al., 2011; Eskow Jaunarajs et al., 2015; Scarduzio et al., 2017; Richter et al., 2019). In accordance with this hypothesis, anticholinergics, such as trihexyphenidyl, are successfully used for treatment in clinical practice (Jankovic, 2013; Maltese et al., 2014). It is assumed that these cholinergic alterations are not caused by changes at the expression level but are caused due to the receptor function (Richter et al., 2019). To further elucidate this assumption, gene expression of the different muscarinic and nicotinic acetylcholine receptor subunits and their function should be investigated in future studies.

To further investigate the functional THAP1 phenotype, we performed whole-cell patch-clamp recordings of MSNs. In our previous MSN studies (Stanslowsky et al., 2016; Capetian et al., 2018), mPSCs were blocked by the GABA_*A*_ receptor antagonist bicuculline, indicating that the afferent synaptic input was predominantly GABAergic. Because morphometric analysis of GABAergic synapses was similar in THAP1 MSNs and healthy controls (Figure 2J), the reduction of synaptic activity, as indicated by a significantly lower frequency of mPSCs in THAP1 MSNs, may rather be the consequence of an impaired afferent GABAergic transmission induced by downregulation of GABA_*A*_ receptor subunits (predominantly α2). In addition, the receptor activation level by different concentrations of GABA may also have influenced the direction of action of presynaptic GABA_*A*_ receptors. It was shown that low levels of presynaptic receptor activation enhance transmitter release, whereas higher levels of activation inhibit release at the same synapses (Khatri et al., 2019). An insufficient afferent, inhibitory GABAergic transmission after neuronal maturation could explain the elevated generation of action potentials in THAP1 MSNs leading to a hyperexcitability phenotype. The functional outcome with an enhanced activity of THAP1 MSNs might eventually result in excessive GABAergic inhibition of striatal MSN projection targets.

This presumption is in line with other studies that show alterations in synaptic transmission due to *THAP1* mutations (Zakirova et al., 2018; Frederick et al., 2019; Cheng et al., 2020). In mouse brain, *Thap1* mutations or deletions led to impaired synaptic plasticity among other dysregulations in pathways of mitochondrial function and the development of neuronal projections (Zakirova et al., 2018). Further, *THAP1* missense mutations in human neuronal cell models showed dysregulation of target genes related to synaptic function and impaired mitochondrial enzyme activity (Cheng et al., 2020). Phenotypical consequences of a loss-of-function mutation in mice revealed alterations in gene expression of cellular pathways for nervous system development, cytoskeleton, synaptic transmission, and gliogenesis (Frederick et al., 2019).

Synaptic function abnormalities were also reported in other isolated dystonia types, including generalized torsion DYT-TOR1A dystonia (Yokoi et al., 2013). Besides this, it was shown in mice that a *Tor1A* mutation led to a change in the calcium dynamics, a hypercholinergic state, and a disinhibition of the GABAergic synaptic activity in medium spiny neurons (Sciamanna et al., 2009; Iwabuchi et al., 2013; Scarduzio et al., 2017). Because the promotor of TOR1A contains two THAP1-binding sequences (Gavarini et al., 2010; Kaiser et al., 2010), it is tempting to speculate that some of the pathophysiological changes seen in DYT-TOR1A dystonia could similarly occur in DYT-THAP1 dystonia.

## Conclusion

In conclusion, the differentiation of patient-derived iPSCs toward MSNs provided a feasible *in vitro* model of DYT-THAP1 dystonia and gave insights into the functional phenotype with altered intracellular calcium dynamics, increased calcium amplitudes after application of acetylcholine, reduced GABA-evoked calcium signals, inhibited synaptic activity and enhanced postsynaptic generation of action potentials. According to our quantitative PCR data, these functional changes may be related to the downregulation of a GABA_*A*_ receptor α2 subunit in THAP1 MSNs. However, the conclusions have to be viewed with caution due to the limited number of analyzed iPSC-derived neuronal lines and the divergent clinical phenotypes of the THAP1 mutation carriers. Further experiments in human stem cell models of DYT-THAP1, including the generation of isogenic control lines as well as the application of different α2 subunit-specific GABA_*A*_ receptor agonists, might be beneficial to get a more comprehensive understanding of the pathophysiological mechanisms underlying this genetic dystonia. Nevertheless, our data potentially contribute to the development of novel treatment strategies (e.g., α2 subunit-specific GABA_*A*_ receptor agonists) for DYT-THAP1 dystonia.

## Data Availability Statement

The original contributions presented in the study are included in the article/Supplementary Material, further inquiries can be directed to the corresponding author/s.

## Author Contributions

SSt, AK, and FW design the study, carry out the study, and write the first draft of the manuscript. FW, PS, MK, and AH revise it critically for important intellectual content. SSt concept and design, data acquisition (medium spiny neurons, calcium imaging, and expression analysis), data analysis (calcium imaging, expression analysis, electrophysiology), and data interpretation (calcium imaging, electrophysiology, and expression analysis), and the manuscript writing. AK: concept and design, data acquisition and analysis (medium spiny neurons and calcium imaging), data interpretation (electrophysiology, calcium imaging, and expression analysis), and the manuscript writing. FW: concept and design, data acquisition (electrophysiology), data interpretation (electrophysiology, calcium imaging, and expression analysis), and the manuscript writing. HB, KL, and PS: data acquisition and interpretation (DYT-THAP1, iPSCs). HG and AH: data acquisition and interpretation (control, iPSCs). LH: data analysis (electrophysiology). TG and NK: data acquisition (medium spiny neurons). MK: data interpretation (electrophysiology, calcium imaging, and expression analysis). All authors contributed to the article and approved the submitted version.

## Conflict of Interest

The authors declare that the research was conducted in the absence of any commercial or financial relationships that could be construed as a potential conflict of interest.

## Footnotes

1 http://mdsgene.org
