# Supplementary material for: Reduced Expression of GABAA Receptor Alpha2 Subunit Is Associated With Disinhibition of DYT-THAP1 Dystonia Patient-Derived Striatal Medium Spiny Neurons
Source: Front Cell Dev Biol. 2021 May 21;9:650586. doi: 10.3389/fcell.2021.650586 (PMC8176025; doi:10.3389/fcell.2021.650586)
Supplement: Supplementary file 1 [file Table_1.DOC]

**Supplementary Table 1.** Quantitative real-time PCR analysis of selected and reference genes for iPSCs and MSNs after 70 days of differentiation *in vitro*. Primer sequences and amplification product in base pairs (bp) are given.

| Targets | Primer sequence (forward; reverse) | Product (bp) |
| --- | --- | --- |
| GAD67  FOXP1  CTIP2  TUBB3  MAP2  SST  B2M  GAPDH  β-actin | AGATCAACAAATGCCTGGAACTGGC;  GAGCCACCTTGTGTAGCTTTTCCC  CCACGTGGAAGAATGCAGTGCG;  GCATTGAGAGGTGTGCAGTAGGC  CTCCGAGCTCAGGAAAGTGTC;  TCATCTTTACCTGCAATGTTCTCC  AGTGATGAGCATGGCATCGACCC;  GGCACGTACTTGTGAGAAGAGGC  CAGGCAAAGGACAAAGTCTCTGACG;  CGCCGAGGAGGGAGAATGGAGG  GAGATCTGCTAACTCAAACCCGGC;  TCGCTGAAGACTTGGAGGATTAGGG  TGCCTGCCGTGTGAACCATGT;  TGCGGAATCTTCAAACCTCCATGA  AGCCACATCGCTCAGACACCAT;  CAGGCGCCCAATACGACCAAAT  CATGTACGTTGCTATCCAGGC;  CTCCTTAATGTCACGCACGAT | 183  193  129  110  92  180  97  71  250 |

**Supplementary Table 2.** Quantitative real-time PCR analysis of GABA_A_ receptor subunit expression in MSNs after 70 days of differentiation *in vitro*. Primer sequences and amplification product in base pairs (bp) are given (Wegner et al., 2008).

| GABA_A_ receptor subunit | Primer sequence (forward; reverse) | Product (bp) |
| --- | --- | --- |
| α1  α2  α3  α4  α5  α6  β1  β2  β3  γ1  γ2  γ3  δ | tgcagcttggagacaggatt;  tgaaccatcttccccctctt  agaggatggacttgggatgg;  aagattcggggcataattgg  cacaagtgtcgttctggctca;  tggcactgatactcaaggtggt  tccggttttcatgcaaaggt;  cttcattaaggataagccagtggaa  ggtgtccttttggctgaacc;  gccactttgggcagagagtt  tttcccaggtgtctttctgga;  ggcactgatgctcaaagtgg  atgcatctgcagccagagtc;  agggatctttggcagggtct  cccaaaccaaatgtcactgc;  tggaactgtcaacttgcttcaaa  attgaaaggcgccatgtttt;  gggttggtcctagggagagg  ggagatgggggatgataggc;  atcccttccacccaacacac  ttgtcgaacaggagcttgga;  gaaggcagtggggaagaaga  aaccaaccaccacgaagaaga;  cctcatgtccaggagggaat  gtctttgctctgcaggatcg;  ccaggccaaggctttatttc | 97  117  99  100  117  101  95  90  104  105  91  113  124 |

**Supplementary Table 3.** Quantitative real-time PCR analysis of voltage-gated Ca^2+^-channel subunit expression in MSNs after 70 days differentiation *in vitro*. Primer sequences and amplification product in base pairs (bp) are given.

| Voltage-gated Ca^2+^-channel subunit | Primer sequence (forward; reverse) | Product (bp) |
| --- | --- | --- |
| Ca_v_ 1.2 (L-type)  Ca_v_ 1.3 (L-type)  Ca_v_ 2.1 (P-type)  Ca_v_ 2.2 (N-type)  Ca_v_ 2.3 (R-type)  Ca_v_ 3.1 (T-type)  Ca_v_ 3.2 (T-type)  Ca_v_ 3.3 (T-type) | CATTTGACGCCTTGATTGTTGTGGG;  GTATGTTCAGCTGGGTTTACCTCGG  CGGACCCCGTCCTCGAAGGA;  CCTACGCGGATCGGGTTGGT  CCAGAAACTTGCCCTACAGAAAGCC;  CGGGTCCATTTCGTTATACAGGGC  TGCTGTTCAGGAGCGCCACG;  CGGTGGCATTGGCCTGCTCA;  GTGGCCCTGGGGTTCATCTTCCATA;  CAGGATGCCACTGAGGACCACGA;  TCAGCCTCCCCCTGAGCGTG;  TTCTGCAGGACCGCATGCCG  GTCACTCTGCTGCTGGATACGC;  TCAGGTTGTTGTTCCTGACAAAGGC  ATCGACTACACCCTGTGCTTCCG;  GACGTAGTCGAAGAGTTTGTGGGC | 73  111  196  93  90  111  160  162 |
